# Orthorexia nervosa and healthy orthorexia as new eating styles

**DOI:** 10.1371/journal.pone.0219609

**Published:** 2019-07-10

**Authors:** Friederike Barthels, Juan R. Barrada, María Roncero

**Affiliations:** 1 Heinrich-Heine-University Düsseldorf, Institute of Experimental Psychology, Department of Clinical Psychology, Düsseldorf, Germany; 2 Universidad de Zaragoza, Teruel, Spain; 3 Universitat de València, Valencia, Spain; University of Lleida, SPAIN

## Abstract

It was recently proposed that healthy orthorexia (HeOr) and orthorexia nervosa (OrNe) should be differentiated. The aim of the present study was to analyze whether the two dimensions of orthorexia can be considered new eating styles or basically equivalent to restrained eating behavior. Two samples of university students (sample 1, *n* = 460; sample 2, *n* = 509) completed the Teruel Orthorexia Scale (TOS), the Dutch Eating Behavior Questionnaire (DEBQ), and the Positive and Negative Affect Schedule (PANAS). Factor analysis with the TOS and DEBQ items together revealed an adequate fit for the preexisting five-factor solution (TOS: OrNe and HeOr; DEBQ: Restrained Eating, Emotional Eating, and External Eating). This result points out that these factors are conceptually distinguishable. Moreover, we tested whether the different eating styles presented different patterns of correlations with gender, body mass index (BMI), and age, and whether OrNe and HeOr predicted Positive and Negative Affect after controlling for Restrained, Emotional, and External Eating. Whereas Restrained and Emotional Eating were higher for women and increased with BMI in both samples, HeOr and OrNe presented much lower associations with these variables. OrNe was positively related to Negative Affect and negatively to Positive Affect, whereas HeOr was positively related to Positive Affect. Again, this result supports the assumption that OrNe is a new variant of disordered eating, whereas HeOr could possibly be seen as a protective behavior.

## Introduction

Using a combination of the Greek words “orthós”–meaning correct–and “órexis”–signifying appetite–, orthorexia has been described as a new eating style. From the same etymological origin of orthorexia, it is clear that an interest in eating right or healthy should not be associated with a problematic approach to food. Most of the attention has been paid to the problematic component of orthorexia: orthorexia nervosa (OrNe). Barrada and Roncero [1] showed that orthorexia includes, in addition to the pathological dimension of OrNe, a non-pathological interest in healthy eating, which they called Healthy Orthorexia (HeOr). Up to now, orthorexia and OrNe have been considered to be basically equivalent.

To define orthorexic eating behavior and its symptoms, several proposals for diagnostic criteria have been made [2]. It has been noted that, "at present there is no universally shared definition of OrNe, and the diagnostic criteria are under debate" [3] (p. 210). Key elements of OrNe are "(a) obsessive focus on dietary practices believed to promote optimum well-being through healthy eating (with inflexible dietary rules, recurrent and persistent preoccupations related to food, compulsive behaviors); and (b) consequent, clinically significant, impairment (e.g. medical or psychological complications, great distress, and/or impairment in important areas of functioning)" [3] (p. 210). Some authors additionally mention that orthorexic symptoms, such as preoccupation and concern about eating impure or unhealthy foods [4], compulsive behavior [2], and overvalued ideas about the effectiveness and potential health benefits of foods [5], are crucial in identifying health-conscious eating behavior as a pathological condition. However, until now, there has not been enough empirical evidence about whether orthorexic eating behavior is a disorder of clinical relevance [3,5,6]. Considering the other elements of orthorexia, key elements of HeOr are a "healthy interest in diet, healthy behavior with regard to diet, and eating healthily as part of one’s identity" [7] (p. 2).

Most studies on orthorexia have been conducted using the ORTO-15 [8], a questionnaire widely criticized for its poor psychometric quality [9–11]. Recently, alternative instruments to the ORTO-15 have been developed, such as the Duesseldorf Orthorexia Scale (DOS) [12] (which was recently published in a Chinese [13], an English [14], and a Spanish version [15]) or the Teruel Orthorexia Scale (TOS) [1], a multidimensional measure of orthorexia. After reducing the initial 31-item version, the TOS is currently a brief scale consisting of 17 items with adequate psychometric properties. The TOS measures both OrNe and HeOr. OrNe and HeOr correlate at around .40–.50. Whereas OrNe has positive correlations with different measures of mental discomfort, for healthy orthorexia these correlations are null or negative, especially when controlling the effects of the pathological variant of orthorexia [1].

However, the OrNe scores presented correlations of .67 with the dimensions of diet and bulimia [1] measured with the Eating Attitudes Test-26 [16]. Barthels, Meyer, and Pietrowsky [17] also found a correlation of .40 between the DOS scores and the Restrained Eating Scale [18] scores in a sample of vegan and vegetarian individuals. Depa et al. [7] found that OrNe and Weight Control motives for food-choice had a correlation of .62. These results raise a question about the possible differentiation between OrNe and other eating styles such as dieting and restrained eating.

Two eating styles can be considered the most relevant theoretically [19]: Restrained eating and emotional eating. Restrained eating [20] is defined as the restriction of food intake in order to control body weight, and it refers predominantly to the amount of food. Emotional eating [21] focuses on eating in response to negative emotions as an atypical response to distress, with the typical response being refraining from eating [22]. These two dimensions have been found when different questionnaires related to eating behaviors have been factor-analyzed, where the avoidant restrictive pattern is clearly differentiated from the more emotional binge-purge pattern [23,24].

Considering (a) the overlap between OrNe and restrained eating, (b) the novelty of HeOr as a different dimension of orthorexia, and (c) previous attempts to introduce new eating styles that are, to a large degree, the same as previously considered ones (e.g., [23,25] for the case of intuitive eating; [24] for food-addiction), we believe that further evidence is needed about the relationship between orthorexia and restrained and emotional eating. The main aim of the present study was to clarify whether orthorexia represents a new eating style or should be considered part of a previously defined eating style, mainly restrained eating. Before addressing the main objective, a set of analyses were performed to verify the existence of the two factors found in the TOS, i.e. OrNe and HeOr, because the reduced 17-item version has not yet been tested with an independent sample. Moreover, the internal structures of the Dutch Eating Behavior Questionnaire (DEBQ) and the Positive and Negative Affect Schedule (PANAS) were also tested. Then, we analyzed whether the factors of orthorexia and the three measured eating styles (restrained, emotional, and, additionally, external eating; external eating focuses on eating in response to food-related sensory cues, such as the sight, smell, and taste of food, regardless of the internal state of hunger and satiety [26]) could be differentiated.

Therefore, two sets of analyses were conducted. First, we analyzed whether orthorexia could be differentiated from emotional eating, food restriction, and external eating. To achieve this aim, a factor analysis was performed, at the item level, of questionnaires assessing orthorexia and the other eating styles, in order to discover whether OrNe or HeOr collapsed in any DEBQ factor. If restrained eating, which focuses on *how much is eaten* and its impact on weight, was basically the same as orthorexia, which focuses on *what is eaten* and its relationship with health, at least two theoretical factors would group together. If all the expected factors are recovered, with each dimension clearly defined, this could provide further evidence that orthorexia represents a new eating style.

The second set of analyses was performed to test whether OrNe and HeOr were differentially related to additional variables. First, basic sociodemographic information (gender, body mass index–BMI–, and age) was considered. If orthorexia dimensions are redundant with respect to the other eating styles, they will present the same relations with these personal characteristics. Second, we tested whether both orthorexia dimensions were related to negative affect and positive affect, even after controlling for the other eating styles. Negative affect reflects emotional distress and includes moods such as fear, sadness, anger and guilt; positive affect is related to experiencing positive mood, with feelings such as joy, interest, enthusiasm, and alertness [27]. If both dimensions of orthorexia tap contents already considered by other eating styles, HeOr and OrNe would not be related to negative and positive affect after controlling for the other eating styles. A "direct correspondence between internalizing and negative affectivity" (p. 461) [28] has been noted. Eating pathology is considered a subfactor of internalizing disorders, and so, clearly, the relationship between affect and orthorexia should be explored. Previous research has shown that OrNe and negative affect are positively correlated, *r* = .28 [1], but this relationship has not been tested when other eating styles are taken into account, and positive affect has not been considered.

In summary, the aim of the present study is to investigate whether OrNe and HeOr are better conceptualized as preexisting eating styles, such as restrained or emotional eating, or should be considered new eating styles.

## Method

### Participants and procedure

The present study formed part of a more comprehensive project carried out in a medium-size Spanish university, the goal of which was to determine key correlates of orthorexia. We approached the participants through the e-mail distribution lists of the University of Zaragoza (Spain). Each student registered on the lists whose administrators gave access to the corresponding information received an e-mail with the goal of the study, contact information of the principal investigator, participation conditions, and a link to access the survey. Only those who accepted the informed consent could gain access. This procedure was approved by the Ethics Review Board for Clinical Research of the region (C.P.-C.I. PI18/340).

#### Sample 1

The initial sample was made up of 575 participants between 17 and 68 years old (M = 23.20, SD = 6.52). Four inclusion criteria were employed: (1) being a resident in Spain (seven participants excluded); (2) currently studying at the university (seven participants excluded); (3) age between 18 and 26 years old, based on criteria from previous studies with university samples (e.g., [29–31]; 78 participants excluded); and (4) correctly answering a control question (see below; 23 participants excluded). After applying these criteria, the final sample comprised 460 participants. Of them, 375 (81.5%) were women and 85 (18.5%) men. Mean age was 21.12 years (SD = 2.19). Data were collected in December 2016. The present sample was used in Depa et al. [7], but in that study, other variables and research questions were considered (relationship between orthorexia and food-choice motives).

#### Sample 2

The initial sample was made up of 635 participants between 18 and 66 years old (M = 23.73, SD = 7.01). Following the same inclusion criteria as for sample 1, except for the control question, 0, 49, and 77 participants were excluded. After applying these criteria, the final sample comprised 509 participants. Of them, 417 (81.9%) were women; 91, men (17.9%); and 1 (0.20%), other sex/gender. Mean age was 21.35 years (SD = 2.09). Data were collected in May and June 2018.

### Measures

#### Sociodemographic data

Participants provided information about their gender (sample 1: women or men; sample 2; women, men, or other), age, and education level. They also reported their weight (to the nearest kilogram) and height (to the nearest centimeter).

#### Teruel Orthorexia Scale (TOS) [1]

This scale assesses orthorexia in two separate dimensions: HeOr (nine items; e.g., "I mainly eat foods that I consider to be healthy") and OrNe (eight items; e.g., "Thoughts about healthy eating do not let me concentrate on other tasks"). Responses are provided on a 4-point scale ranging from 0 = *Completely disagree* to 3 = *Completely agree*. For sample 1/sample 2, Cronbach's alpha values for HeOr were .85/.87; for OrNe, .83/.82.

#### (Full and Short) Dutch Eating Behavior Questionnaire (DEBQ) [32]

Sample 1 answered the full version of the DEBQ (33 items), and sample 2 answered the short version (19 items) [33]. This scale assesses eating styles in three separate dimensions: Restrained Eating (10 or seven items; e.g., "Do you deliberately eat less in order to not become heavier?"), Emotional Eating (13 or six items; e.g., "Do you have the desire to eat when you are irritated?"), and External Eating (10 or six items; e.g., "Do you eat more than usual when you see others eating?"). Although in some studies based on total scores Emotional Eating and External Eating have loaded in the same factor, factor analyses at the item level revealed that the two dimensions can be separated [34–36]. Responses are provided on a 5-point scale ranging from 1 = *seldom* to 5 = *very often*. We used the Spanish version [36]. For sample 1/sample 2, Cronbach's alpha values for Restrained Eating were .91/.84; for Emotional Eating, .94/93; and for External Eating, .85/.79.

#### Positive and Negative Affect Schedule (PANAS) [27]

This scale was developed to assess affect in two separate dimensions: Negative Affect (10 items; e.g., "Nervous") and Positive Affect (10 items; e.g., "Enthusiastic"). Although there is some controversy about the internal structure of the PANAS, with some authors defending the convenience of splitting the Negative Affect factor into two dimensions [37,38], the most common use of this questionnaire is still as it was theoretically constructed. Responses are provided on a five-point scale ranging from 1 = *very slightly or not at all* to 5 = *extremely*. We used two different Spanish adaptations: Joiner, Sandin, Chorot, Lostao, and Marquina [39] for sample 1 and Sandín et al. [40] for sample 2. For sample 1, participants were instructed to answer by considering their feelings in the past week; in sample 2, they were asked to answer by considering how they usually felt. For sample 1/sample 2, Cronbach's alpha values for Negative Affect were .85/.86; for Positive Affect, .88/.84.

#### Control question

In order to check whether the participants paid enough attention to the wording of the items, we introduced an item asking the participants to respond with a specific alternative. Only sample 1 performed this check.

### Analyses

We followed the same four steps to analyze the data for both samples. First, we tested the dimensional structure of the TOS scores, the DEBQ scores (full version and short version), and the PANAS scores separately. For the TOS scores, we tested an exploratory structural equation model (ESEM) [41,42] with two factors [1]. For the DEBQ, we tested a three-factor ESEM with the correlated uniquenesses described in previous publications [35,36]. For the PANAS, we tested a two-factor ESEM with the correlated uniqueness described by Crawford and Henry [43] and based on Zevon and Tellegen [44], which have been applied to the Spanish version of the scale [45].

Second, we analyzed the factor structure of both the items on the TOS (two theoretical factors) and the items on the DEBQ (three theoretical factors). All the items were simultaneously submitted to an ESEM analysis. If the TOS and the DEBQ are assessing conceptually distinguishable—albeit related—constructs, a solution with five factors should show an adequate fit and a clear structure.

In the third step, we modeled the responses to the TOS and DEBQ items simultaneously with gender, BMI, and age included in the model. For this model, in sample 2 we excluded the participant who did not identify as either a woman or a man.

In the fourth step, we modeled the responses to the items from all the questionnaires simultaneously. The TOS and DEBQ scores defined a first set of factors, whereas the PANAS scores defined a second set. This means that the TOS and DEBQ items could not show cross-loadings with the PANAS items. In this structural model, affect factors were predicted by factors from the five different eating styles.

Goodness of fit of all the derived models was assessed with the common cut-off values for the fit indices [46]: CFI and TLI with values greater than .95 and RMSEA less than .06 are indicative of a satisfactory fit. It should be noted that these cut-offs were developed for confirmatory factor analysis with continuous responses, and so these values should be interpreted with caution. The authors are not aware that specific cut-offs have been proposed for exploratory analyses with categorical variables.

For all the models, the WLSMV estimator was used. By using this estimator, we were able to maintain the categorical nature of the responses [47]. We used target rotation. As described by Asparouhov and Muthén [41], "conceptually, target rotation can be said to lie in between the mechanical approach of EFA [exploratory factor analysis] rotation and the hypothesis-driven CFA [confirmatory factor analysis] model specification. In line with CFA, target loading values are typically zeros representing substantively motivated restrictions. Although the targets influence the final rotated solution, the targets are not fixed values as in CFA, but zero targets can end up large if they do not provide good fit" (p. 409). Target rotation has previously been used in the area of eating styles with the DEBQ items [25]. For all the factor models, we interpreted the standardized solution (STDYX solution in MPlus). The correlation between gender and the different eating styles was transformed to Cohen's *d* [48] to facilitate its interpretation.

All the latent models were estimated with Mplus 7.4 [49]. The rest of the analyses were performed with R 3.5.2 [50]. We used the packages psych version 1.8.12 [51] and MplusAutomation version 0.7 [52]. No missing data were present in our database. The open database and code files for these analyses are available at the Open Science Framework repository (https://osf.io/kagxy/).

## Results

### Internal structure of the different instruments

The model fit of the different models is presented in Table 1. Regarding the TOS structure, for both samples, the CFI was satisfactory (CFI = .960/.973 for sample 1/sample 2), whereas the TLI for the first sample was slightly below the intended threshold (TLI = .947/.965), and the RMSEA of both samples (RMSEA = .070/.062) was slightly above the cutoff. The item loadings for the TOS are shown in Table 2. The pattern of loadings reflects the bidimensional structure of orthorexia, although three out of 34 cross-loadings were above 0.30 (maximum = 0.36).

**Table 1:** Goodness of fit indices for the different models.

Models	*χ*^2^	*df*	CFI	TLI	RMSEA
First sample					
M1.1. TOS	335.2	103	.960	.947	.070
M1.2. Full DEBQ	1010.7	423	.972	.965	.055
M1.3. PANAS	464.2	138	.957	.941	.072
M1.4. TOS & Full DEBQ	1698.0	976	.969	.961	.040
M1.5. TOS & Full DEBQ & Sociodemographics	1868.2	1111	.968	.961	.038
M1.6. TOS & Full DEBQ & PANAS	3072.4	2104	.964	.958	.032
Second sample					
M2.1. TOS	302.3	103	.973	.965	.062
M2.2. Short DEBQ	472.9	117	.977	.966	.077
M2.3. PANAS	628.8	138	.940	.917	.084
M2.4. TOS & Short DEBQ	947.0	460	.975	.966	.046
M2.5. TOS & Short DEBQ & Sociodemographics	1086.5	553	.974	.965	.044
M2.6. TOS & Short DEBQ & PANAS	2009.3	1308	.970	.964	.032

Notes: df = degrees of freedom; TLI = Tucker-Lewis index; CFI = comparative fit index; RMSEA = root mean square error of approximation; TOS = Teruel Orthorexia Scale; DEBQ = Dutch Eating Behavior Questionnaire; PANAS = Positive and Negative Affect Schedule. All *p*-values for the *χ*^2^ test were < .001.

**Table 2:** Item loadings for the Teruel Orthorexia Scale.

	HeOr	OrNe
T01. I feel good when I eat healthy food.	**0.61** / **0.65**	0.07 / 0.15
T02. I spend a lot of time buying, planning and/or preparing food so my diet will be as healthy as possible.	**0.74** / **0.74**	–0.06 / 0.08
T03. I believe that the way I eat is healthier than that of most people.	**0.88** / **0.91**	–0.23 / –0.21
T04. I feel guilty when I eat food that I do not consider healthy.	0.00 / –0.01	**0.81** / **0.78**
T05. My social relations have been negatively affected by my concern about eating healthy food	0.25 / 0.23	**0.66** / **0.64**
T06. My interest in healthy food is an important part of the way I am, of how I understand the world	**0.66** / **0.75**	0.21 / 0.20
T07. I'd rather eat a healthy food that is not very tasty than a good tasting food that isn't healthy	**0.70** / **0.69**	0.13 / 0.12
T08. I mainly eat foods that I consider to be healthy	**0.86** / **0.94**	–0.17 / –0.20
T09. My concern with healthy eating takes up a lot of my time	0.25 / **0.36**	**0.58** / **0.52**
T10. I am concerned about the possibility of eating unhealthy foods	0.10 / 0.23	**0.59** / **0.55**
T11. I don't mind spending more money on food if I think it is healthier.	**0.55** / **0.55**	0.09 / 0.07
T12. I feel overwhelmed or sad if I eat food that I consider unhealthy	–0.03 / –0.02	**0.90** / **0.87**
T13. I prefer to eat a small quantity of healthy food rather than a lot of food that may not be healthy	**0.41** / **0.43**	**0.32** / 0.26
T14. I avoid eating with people who do not share my ideas about healthy eating	0.17 / **0.31**	**0.61** / **0.31**
T15. I try to convince people from my environment to follow my healthy eating habits	**0.52** / **0.58**	0.08 / 0.09
T16. If, at some point, I eat something that I consider unhealthy, I punish myself for it	–0.18 / –0.18	**0.93** / **0.94**
T17. Thoughts about healthy eating do not let me concentrate on other tasks	–0.16 / –0.25	**1.00** / **0.97**

Notes: HeOr = Healthy Orthorexia; OrNe = Orthorexia Nervosa. Shaded cells indicate the factor where the item theoretically belongs. Loadings in bold indicate unsigned loadings above |.30|. Underlined loadings indicate cross-loadings above |.30|. The correlation between Healthy Orthorexia and Orthorexia Nervosa was .44/.49 for sample 1/sample 2.

Both versions of the DEBQ presented an adequate fit (CFI = .972/.977, TLI = .965/.966), with the exception of RMSEA for the second sample (RMSEA = .055/.077). The model fit for the PANAS was worse, with five out of six indices showing inadequate values (CFI = .957/.940, TLI = .941/917; RMSEA = .072/.084). However, the structure indicated the recovery of two clear factors with the expected theoretical interpretation.

### Analysis of relationship between Orthorexia and Emotional Eating, Restrained Eating, and External Eating

A factor analysis at the item level was performed with the TOS and DEBQ to analyze whether the five-factor structure (two from the TOS and three from the DEBQ) could be recovered, thus differentiating their factors. For both samples, model fit was satisfactory (CFI = .969/.975, TLI = .961/.966, RMSEA = .040/.046). The item loadings and inter-factor correlations can be seen in Table 3. With regard to the item loadings, four main aspects should be noted. First, the distribution of items per factor was clear, and the five-factor structure was recovered. Second, Item 13 from the TOS ("I prefer to eat a small quantity of healthy food rather than a lot of food that may not be healthy") presented a low loading (λ) in the intended factor (HeOr; λ = 0.28/0.33), with loadings of similar sizes in OrNe and Restrained Eating. Third, the loadings of DEBQ Item 14 ("Do you watch exactly what you eat?") in the intended factor were small (Restrained Eating; λ = 0.16/0.14), whereas they were higher in HeOr (λ = 0.45/0.49). Fourth, DEBQ Item 3 ("Desire to eat when nothing to do…") loaded above 0.30 in both Emotional and External Eating, as previously reported [36].

**Table 3:** Item loadings and interfactor correlations for the Teruel Orthorexia Scale and the Dutch Eating Behavior Questionnaire.

	Factor Loadings				
	HeOr	OrNe	Res	Emot	Ext
T01. I feel good when I eat healthy food.	**0.61** / **0.62**	0.02 / 0.19	0.14 / –0.07	–0.06 / 0.10	0.07 / –0.07
T02. I spend a lot of time buying, planning and/or preparing food so my diet will be as healthy as possible.	**0.73** / **0.75**	–0.08 / 0.09	0.08 / 0.01	0.02 / 0.09	–0.06 / –0.01
T03. I believe that the way I eat is healthier than that of most people.	**0.86** / **0.91**	–0.16 / –0.18	–0.01 / 0.01	–0.05 / –0.03	0.04 / 0.09
T06. My interest in healthy food is an important part of the way I am, of how I understand the world	**0.62** / **0.72**	0.27 / 0.28	–0.04 / –0.07	0.00 / 0.05	–0.09 / –0.06
T07. I'd rather eat a healthy food that is not very tasty than a good tasting food that isn't healthy	**0.58** / **0.62**	0.22 / 0.23	–0.01 / –0.02	–0.04 / –0.07	–0.20 / –0.10
T08. I mainly eat foods that I consider to be healthy	**0.80** / **0.93**	–0.14 / –0.22	0.07 / 0.10	–0.06 / –0.02	–0.03 / 0.00
T11. I don't mind spending more money on food if I think it is healthier.	**0.56** / **0.51**	0.18 / 0.06	–0.12 / 0.11	0.04 / –0.01	0.04 / –0.07
T13. I prefer to eat a small quantity of healthy food rather than a lot of food that may not be healthy	0.28 / **0.33**	0.28 / 0.29	0.24 / 0.23	–0.09 / –0.24	–0.24 / –0.14
T15. I try to convince people from my environment to follow my healthy eating habits	**0.50** / **0.58**	0.20 / 0.11	–0.09 / 0.06	–0.10 / –0.12	0.16 / 0.18
T04. I feel guilty when I eat food that I do not consider healthy.	–0.01 / –0.01	**0.68** / **0.71**	0.23 / 0.13	–0.04 / –0.01	0.05 / 0.03
T05. My social relations have been negatively affected by my concern about eating healthy food	**0.31** / 0.28	**0.57** / **0.43**	0.06 / 0.2	0.13 / 0.14	–0.07 / –0.06
T09. My concern with healthy eating takes up a lot of my time	0.29 / **0.37**	**0.61** / **0.50**	–0.09 / 0.00	0.10 / 0.04	–0.02 / 0.02
T10. I am concerned about the possibility of eating unhealthy foods	0.08 / 0.16	**0.70** / **0.74**	–0.10 / –0.18	–0.08 / –0.06	0.13 / 0.03
T12. I feel overwhelmed or sad if I eat food that I consider unhealthy	–0.04 / –0.04	**0.82** / **0.87**	0.16 / 0.01	–0.06 / –0.01	0.05 / 0.05
T14. I avoid eating with people who do not share my ideas about healthy eating	0.18 / **0.33**	**0.57** / 0.22	0.08 / 0.07	0.01 / 0.08	0.05 / 0.04
T16. If, at some point, I eat something that I consider unhealthy, I punish myself for it	–0.16 / –0.14	**0.76** / **0.75**	0.29 / 0.23	–0.04 / 0.03	0.01 / 0.07
T17. Thoughts about healthy eating do not let me concentrate on other tasks	–0.06 / –0.20	**0.85** / **0.77**	0.08 / 0.15	0.15 / 0.16	–0.03 / –0.02
D04. Eat less than usual when gained weight…	0.02 /–––	–0.08 /–––	**0.85** /–––	–0.01 /–––	–0.01 /–––
D07. Reject food or drinks because worry about weight…	0.00 / 0.02	0.16 / 0.07	**0.77** / **0.81**	0.08 / 0.04	–0.14 / –0.06
D11. Eat less during meal times…	–0.04 /–––	0.04 /–––	**0.81** /–––	0.00 /–––	0.07 /–––
D14. Watch what you eat…	**0.45** / **0.49**	0.24 / 0.21	0.16 / 0.14	0.01 / 0.02	–0.06 / –0.07
D17. Eat food that are slimming…	0.02 / 0.11	0.06 / –0.04	**0.65** / **0.74**	0.10 / 0.03	0.01 / –0.02
D19. Eating less after eating too much…	0.05 / –0.02	0.09 / 0.09	**0.69** / **0.56**	–0.08 / 0.00	0.06 / 0.04
D22. Eat less deliberately . . .	0.06 / 0.04	0.00 / 0.01	**0.91** / **0.81**	–0.03 / 0.06	0.04 / 0.04
D26. Not to eat because watching your weight . . .	–0.04 /–––	0.06 /–––	**0.77** /–––	0.12 /	–0.07 /–––
D29. Try not to eat in evening because watching weight…	–0.23 / –0.26	0.19 / 0.15	**0.61** / **0.62**	0.07 / 0.10	0.02 / 0.04
D31. Take into account weight when eat…	0.13 / 0.08	0.05 / 0.05	**0.77** / **0.80**	0.06 / –0.01	–0.06 / 0.04
D01. Desire to eat when irritated…	0.09 /–––	–0.15 /–––	0.10 /–––	**0.76** /–––	0.05 /–––
D03. Desire to eat when nothing to do…	–0.02 /–––	–0.12 /–––	0.18 /–––	**0.44** /–––	**0.31** /
D05. Desire to eat when you feel depressed…	0.06 / 0.04	–0.13 / –0.10	0.24 / 0.16	**0.76** / **0.86**	0.08 / –0.03
D08. Eating when you feel lonely…	–0.05 /–––	0.19 /–––	0.05 /–––	**0.57** /–––	0.18 /–––
D10. Desire to eat when somebody lets you down…	–0.07 / –0.08	0.14 / 0.13	–0.06 / 0.03	**0.80** / **0.80**	0 / 0.03
D13. Desire to eat when angry…	–0.02 / 0.00	–0.04 / 0.08	–0.02 / –0.11	**0.81** / **0.73**	0.03 / 0.16
D16. Desire to eat when unpleasant…	–0.07 /–––	0.04 /–––	–0.04 /–––	**0.87** /–––	–0.12 /–––
D20. Desire to eat when anxious…	0.11 / 0.07	–0.19 / –0.14	0.11 / 0.15	**0.90** / **0.86**	–0.03 / 0.04
D23. Desire to eat when things go against you . . .	–0.03 / –0.08	0.03 / 0.08	0.00 / –0.02	**0.92** / **0.91**	–0.06 / 0.01
D25. Desire to eat when upset . . .	0.00 /–––	–0.02 /–––	–0.02 /–––	**0.94** /–––	–0.04 /–––
D28. Desire to eat when boring…	0.02 /–––	–0.04 /–––	0.10 /–––	**0.55** /–––	0.27 /–––
D30. Desire to eat when frightened…	–0.11 /–––	0.11 /–––	–0.15 /–––	**0.82** /–––	–0.08 /–––
D32. Desire to eat when disappointed…	–0.04 / –0.05	0.14 / 0.09	–0.16 / –0.06	**0.91** / **0.94**	–0.07 / –0.04
D02. Eat more than usual when tasty…	–0.01 /–––	0.03 /–––	–0.04 /–––	0.14 /–––	**0.58** /–––
D06. Eat more than normal when food is good…	0.08 / –0.04	0.00 / 0.04	–0.04 / –0.04	0.11 / 0.15	**0.60** / **0.56**
D09. Desire to eat eating when delicious…	0.04 / 0.01	–0.07 / –0.03	0.05 / 0.06	–0.12 / –0.10	**0.88** / **0.76**
D12. Eat it immediately when delicious…	–0.10 /–––	0.07 /–––	–0.03 /–––	0.04 /–––	**0.48** /–––
D15. Desire to eat something delicious…	–0.08 / –0.14	0.00 / –0.05	0.06 / 0.12	0.00 / –0.07	**0.57** / **0.66**
D18. Desire to eat when others eating…	–0.01 / 0.16	0.10 / 0.01	–0.04 / –0.08	0.06 / 0.15	**0.60** / **0.65**
D21. Difficult to resist delicious food . . .	–0.04 /–––	–0.01 /–––	0.05 /–––	0.05 /–––	**0.77** /–––
D24. Desire to buy food when bar . . .	–0.05 / –0.06	0.07 / 0.1	–0.09 / 0.00	0.06 / –0.10	**0.62** / **0.82**
D27. Eat more than usual when others eating . . .	–0.02 / 0.15	0.12 / 0.10	–0.05 / –0.04	0.18 / 0.20	**0.48** / **0.50**
D33. Eating when preparing meal…	0.02 /–––	–0.02 /–––	–0.07 /–––	0.02 /–––	**0.46** /–––
	Inter-factor Correlations				
	HeOr	OrNe	Res	Emot	Ext
HeOr					
OrNe	.40 / .46				
Res	.18 / .18	.53 / .60			
Emot	–.22 / .02	.24 / .35	.28 / .35		
Ext	–.25 / .11	–.08 / .06	.03 .03	.47 / .47	

Notes: HeOr = Healthy Orthorexia; OrNe = Orthorexia Nervosa; Res = Restrained Eating; Emot = Emotional Eating; Ext = External Eating. Item numbering starting with T corresponds to the TOS; starting with D, to the DEBQ. Shaded cells indicate the factor where the item theoretically belongs. Loadings in bold indicate unsigned loadings above |.30|. Underlined loadings indicate cross-loadings above |.30|. Exact wording of the DEBQ items cannot be shown due to copyright restrictions.

With regard to the inter-factor correlations, and limiting our attention to the correlations above |.30| in both samples, the factors with the highest overlap were OrNe and Restrained Eating (*r*s = .53/.60), followed by External Eating and Emotional Eating (*r*s = .47/.47) and HeOr and OrNe (*r*s = .40/.46).

### Analysis of relationship between eating styles and gender, BMI, and age

We tested whether the patterns of associations between the five eating styles and the sociodemographic characteristics differed. These results can be seen in Table 4. For both samples, model fit was satisfactory (CFI = .968/.974, TLI = .961/965, RMSEA = .038/.044). With regard to gender, women scored significantly higher than men on Restrained, Emotional, and External Eating (*d*s in the range [0.26, 0.53], but no statistically significant differences were found for the two orthorexia dimensions. In the case of BMI, Restrained and Emotional Eating showed positive and significant correlations for both samples (*r*s in the range [.21, .36]), whereas the other eating styles presented smaller associations that were only significant in one of the two samples. Regarding age, all the correlations could be considered small, with none of them found to be significant in both samples.

**Table 4:** Associations of the different item styles with sociodemographic variables.

	*d*	*r*	
	Gender	BMI	Age
HeOr	0.10 / 0.08	**–.14** /–.05	**.13** / .05
OrNe	–0.13 / –0.11	.08 / **.13**	**–.19** /–.08
Res	**–0.53** / –**0.32**	**.29** / **.36**	–.04 / .00
Emot	**–0.38** / –**0.36**	**.21** / **.25**	–.02 /–.03
Ext	**–0.26** / –**0.30**	.00 /–**.09**	.05 /–.06

Notes: HeOr = Healthy Orthorexia; OrNe = Orthorexia Nervosa; Res = Restrained Eating; Emot = Emotional Eating; Ext = External Eating; *d* = Cohen's d; *r* = Pearson's correlation. Gender is coded with a dummy variable, where 0 = *women* and 1 = *men*. Bold values correspond to statistically significant results, *p* < .05.

### Analysis of the relationship between eating styles and affect

**Fig 1:**
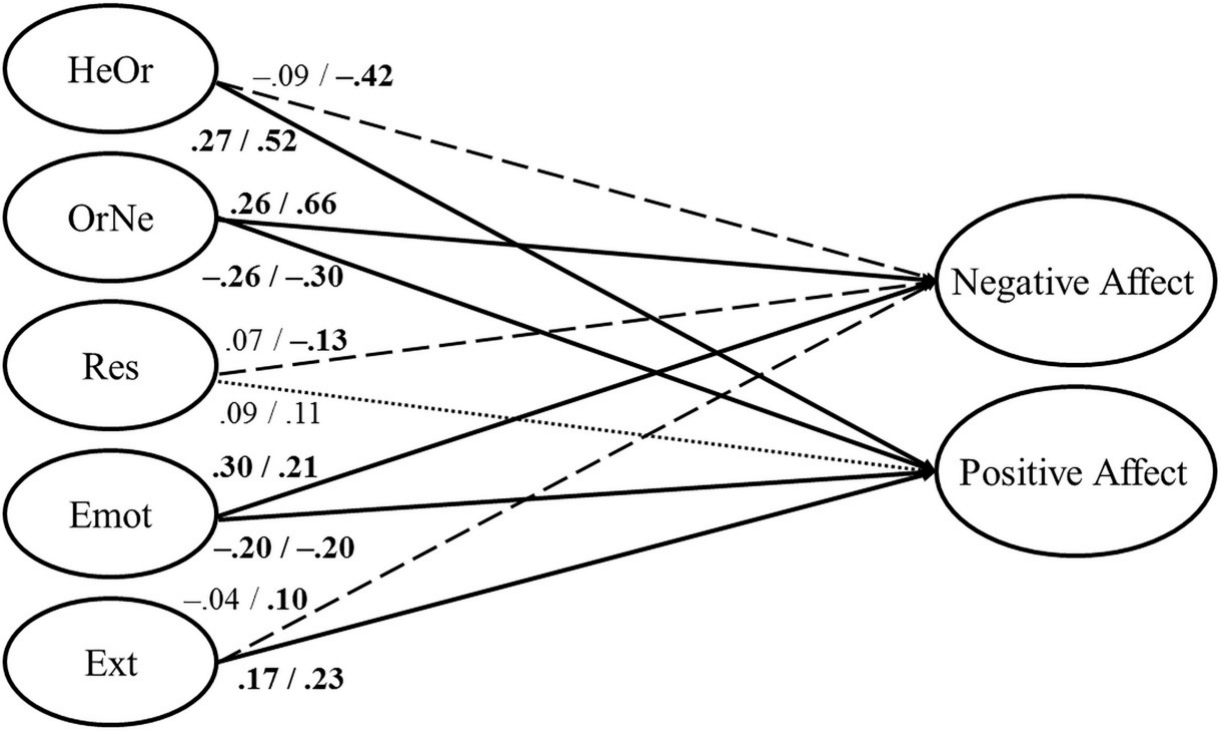
Structural model of the relationships between the five different eating styles and positive and negative affect. Solid arrows correspond to statistically significant (*p* < .05) coefficients in both samples; dashed arrows correspond to statistically significant coefficients in just one of the two samples; dotted arrows correspond to non-statistically significant coefficients in both samples. Bold values correspond to statistically significant coefficients. HeOr = Healthy Orthorexia; OrNe = Orthorexia Nervosa; Res = Restrained Eating; Emot = Emotional Eating; Ext = External Eating.

Finally, we tested whether OrNe and HeOr predicted Positive and Negative Affect, after controlling for Restrained eating, Emotional Eating, and External Eating. For both samples, model fit was satisfactory (CFI = .964/.970, TLI = .958/.964, RMSEA = .032/.032). The representation of this structural model can be seen in Fig 1. Describing only statistically significant coefficients above |.20|, we found that: (1) HeOr was negatively related to Negative Affect in the second sample, β = –.42, and positively related to Positive Affect in both samples, β = .27/.52; (2) OrNe was positively related to Negative Affect, β = .26/.66, and negatively related to Positive Affect, β = –.26/–.30; (3) increases in Emotional Eating were associated with increases in Negative Affect, β = .30/.21, and decreases in Positive Affect, β = –.20/–.20; and (4) increases in External Eating were associated with increases in Positive Affect in the second sample, β = .23. The coefficients for Restrained Eating were small, in the range [–.13, 11]. The percentage of explained variance for Negative Affect was 21.3%/45.4%, and for Positive Affect, 14.0%/22.9%.

## Discussion

Given the novelty of the concept of orthorexia and the controversy about its conceptualization, the main objective of the present study was to analyze whether orthorexia should be considered a preexisting eating style, i.e. restrained eating, emotional eating, or external eating, or be defined as a new eating style.

We started by testing the adequacy of the three questionnaires, with overall satisfactory results, although some minor problems of misfit were detected, mainly for the PANAS in sample 2. We have to note that our model fit (CFI = .940, TLI = .917, RMSEA = .084) is to a large degree equivalent to the model fit reported by Allan et al. [37] (CFI = .95, RMSEA = .03) or by Ortuño-Sierra et al. [38] (CFI = .92, TLI = .91, RMSEA = .07), although these trifactor models are more complex and incorporated correlated uniquenesses that were not defined a priori. In comparison, all the correlated uniquenesses in our model were theoretically predefined [43,44]. Allan et al. [37] reported a correlation between the two subdimensions of Negative Affect in the range of [.75, .92], which indicates that both aspects of Negative Affect (if they are really needed) can be summarized to a large degree by a single dimension. Ortuño-Sierra et al. [38] did not report the inter-factor correlations. These patterns of results cast doubt on the need to incorporate a third factor to correctly model the PANAS scores. From our point of view, the internal structure of the PANAS is still an open topic. From our point of view, part of the controversy will be solved when ESEM models are tested, and not CFA models, because the latter can distort item loadings and inter-factor correlations in the presence of cross-loadings [41,42].

Next, the dependence/independence of orthorexia from restrained eating, emotional eating, and external eating was studied. The factor analysis of the TOS and the DEBQ confirmed that these factors can be separated. Importantly, OrNe and Restrained Eating, although related, are not equivalent. Almost every item loaded in its corresponding factor. Two exceptions were observed: Item 13 from the TOS ("I prefer to eat a small quantity of healthy food rather than a lot of food that may not be healthy") and Item 14 from the DEBQ (regarding the control over *what* you eat). Item 13, which belongs to the OrNe factor (TOS), loaded in the Restrained eating factor (DEBQ). This result seems to make sense, given the wording of the item, which expresses the preference for eating a "small quantity" of healthy food rather than a "larger quantity", but not healthy. Therefore, people with a restrained eating pattern can fail to take into account the nuance of the quality of food and focus only on the quantity. Item 14 of the DEBQ, which belongs to the Restrained Eating factor, also loads in the OrNe factor. This makes sense because the item asks about careful control over *what* you eat. A similar trend was found for this item when it was analyzed with items measuring intuitive eating [25]. In people with orthorexic preoccupations, this control would involve the quality, whereas in restrained eating people, it can also involve the quantity, e. g. the calorie content of the food. An important point of these cross-loadings is that they show that the independence of the factors is not due to a measurement artifact, for example, the fact that the questionnaires were presented on different pages or with different response scales. If this had been the case, we would not have found interpretable cross-loadings. In addition, we found that the TOS and DEBQ factors were correlated. The highest correlations were observed between the OrNe factor and the Restrained Eating factor, as could be expected [1,17].

We studied the relationships between the five identified eating styles and sociodemographic information, namely, gender, BMI, and age. Importantly, the pattern of assocations between Restrained Eating and OrNe differed. Whereas the former was related to gender (higher for women) and positively correlated with BMI, OrNe was not related to any of these variables. This pattern for OrNe [53] and Restrained Eating [35] had been previously reported. Apparently, both orthorexia dimensions were basically unrelated to any of the sociodemographic variables considered.

To further explore the incremental validity of the TOS, we tested whether the OrNe and the HeOr predicted Positive and Negative affect, apart from the other eating styles. Results revealed that the two dimensions of orthorexia present significant regression coefficients. The most powerful associations (i.e., observed in the two samples) were between HeOr and OrNe and Positive Affect and between OrNe and Negative affect. HeOr was associated in a positive direction with Positive Affect, whereas OrNe was associated in a negative direction with Positive Affect and positively with Negative Affect. These results are consistent with the results described by Barrada and Roncero [1]. This pattern of results stresses the idea that, when talking about orthorexia, we should carefully distinguish between OrNe and HeOr: whereas the former is related to psychological distress, the latter is related to well-being.

It is worth noting that the association between HeOr and Negative Affect was significant in one sample, but not in the other. In general, there is a discrepancy between the coefficients of associations between the TOS and PANAS factors in sample 1 and in sample 2. We cannot provide an explanation for this result. It could be due to sampling error; to an unexpected effect of changing the time frame for the PANAS items, as stated in the method section; to the change in the Spanish version of the questionnaire [39,40]; or to the inclusion or not of a control question. However, the discrepancy in the case of the TOS is greater than the one found for the DEBQ factors. Nevertheless, the important point is that the direction of each association is the same in both samples.

The present study has some limitations. First, the sample is composed of university students, which reduces its representativeness of the general population. Moreover, different patterns could be present in clinical samples. The use of non-representative samples is common practice in the research in the area of orthorexia. Second, up until now, the TOS has not been tested with non-Spanish samples. It is necessary to evaluate whether the bidimensional structure of orthorexia and its relationship with other eating styles hold in other cultures/languages. Third, the sample was composed of voluntary participants, which can bias the results in an unknown direction. Fourth, we only used self-report measures, even for height and weight. Fifth, because both samples were extracted from the same university, although 18 months apart, we can expect some overlap in their participants.

Several strengths of the present study should be noted. First, we worked with two samples (combined *n* = 969) in order to cross-validate our main results. Second, we relied on latent variables (factor analyses, structural equation modeling) rather than manifest ones (sum of items). By doing so, we reduced the risk of the results being affected by measurement error [54]. Third, our analyses were based on the item level, which allowed us to detect minor but theoretically relevant and interpretable cross-loadings. We encourage researchers to perform factor analysis at the item level because it can provide relevant information. Factor analysis based on total scores is equivalent to item parceling, a procedure that can distort the correlations between constructs if the combined items are not purely unidimensional [55]. Fourth, we used measures with adequate psychometric properties (TOS, DEBQ, PANAS). An important limitation of the research on orthorexia is the use of the ORTO-15 because it is not very clear what this instrument is measuring [9–11]. Fifth, we considered the bidimensional structure of orthorexia. HeOr is a very new concept that has received very little attention until now.

## Conclusions

Orthorexia shares a key component with restrained eating: self-imposed restriction of allowed food. But orthorexia is focused on food quality and not quantity. This raises the question of whether orthorexia should be conceptualized as essentially equivalent to restrained eating or as a new eating style. The present study provides empirical evidence for the assumption that orthorexic eating behavior is a new variant of eating behavior.

To date, there is no consensus about the nosological classification of orthorexia nervosa. The two orthorexic dimensions cannot both be considered to be related to eating disorders. Whereas HeOr is related to the selection of healthy food (based on its composition) and might serve as a protective factor against emotional distress, OrNe is related to negative affect and the fear of not eating healthily enough. Thus, only OrNe is tapping disordered dimensions. Taking this into account, associations between OrNe and specific characteristics of eating disorders, such as body image issues [1,56–58], drive for thinness [5], and restrained eating behavior [1,17], should be interpreted as evidence for OrNe being part of the eating disorder family. More specifically, this study provides evidence for the assumption that OrNe and restrained eating are distinct constructs because the former is associated with what is eaten and the latter is related to how much is eaten and its impact on weight. In other words, OrNe broadens our conception of restrained eating because we also need to consider restrictions based on healthy food content.

The specific correlational pattern of HeOr is positively linked to positive affect, whereas OrNe is positively linked to negative affect and negatively associated with positive affect. These results provide evidence for the assumption that OrNe is a psychological condition that can cause distress and impairments in everyday life. Because this study analyzed the theoretical constructs of both possible variants of orthorexia, healthy and nervosa, and other eating styles, in a sample of healthy individuals, further studies should investigate the clinical presentation of orthorexic eating behavior in depth.
